# Motivation States for Physical Activity and Sedentary Behavior: Desire, Urge, Wanting, and Craving

**DOI:** 10.3389/fpsyg.2020.568390

**Published:** 2020-11-06

**Authors:** Matthew A. Stults-Kolehmainen, Miguel Blacutt, John B. Bartholomew, Todd A. Gilson, Garrett I. Ash, Paul C. McKee, Rajita Sinha

**Affiliations:** ^1^Bariatric and Minimally Invasive Surgery Program, Yale-New Haven Hospital, New Haven, CT, United States; ^2^Department of Biobehavioral Sciences, Teachers College, Columbia University, New York, NY, United States; ^3^Department of Kinesiology and Health Education, The University of Texas at Austin, Austin, TX, United States; ^4^Department of Kinesiology and Physical Education, Northern Illinois University, DeKalb, IL, United States; ^5^Pain Research, Informatics, Multi-morbidities, and Education (PRIME), VA Connecticut Healthcare System, West Haven, CT, United States; ^6^Center for Medical Informatics, Yale School of Medicine, New Haven, CT, United States; ^7^Department of Psychology, Southern Connecticut State University, New Haven, CT, United States; ^8^Yale Stress Center, Yale School of Medicine, New Haven, CT, United States

**Keywords:** urge for movement, desire, physical activity, exercise, motivation, sedentary activity, motivation states, conceptual analysis

## Abstract

To better explain daily fluctuations in physical activity and sedentary behavior, investigations of motivation are turning from social cognitive frameworks to those centered on affect, emotion and automaticity, such as the Affect and Health Behavior Framework (AHBF), Integrated Framework and Affective-Reflective Theory (ART). This shift has necessitated: (a) re-examination of older theories and their constructs, such as drives, needs and tensions and (b) an inspection of competing theories from other fields that also attempt to explain dynamic changes in health behaviors. The Dynamical Model of Desire, Elaborated Intrusion Theory and others commonly share with AHBF the idea that human behavior is driven strongly by *desires* and/or the similar concepts of wants, urges, and cravings. These *affectively-charged motivation states* (ACMS) change quickly and may better explain physical activity behavior from one moment to the next. Desires for movement predominantly derive from negative but also positive reinforcement. Data from clinical populations with movement dysfunction or psychiatric disorders provides further evidence of these drivers of movement. Those with Restless Legs Syndrome, akathisia, tic disorders and exercise dependence all report strong urges to move and relief when it is accomplished. Motor control research has identified centers of the brain responsible for wants and urges for muscular movement. Models elaborated herein differentiate between wants, desires, urges and cravings. The WANT model (Wants and Aversions for Neuromuscular Tasks) conceptualizes desires for movement and rest as varying by magnitude, approach or avoidance-orientation (wants versus aversions) and as occupying independent dimensions instead of opposite ends of the same axis. For instance, one hypothetically might be in a state of both high desire for movement and rest simultaneously. Variations in motivation states to move and rest may also be associated with various stress states, like freezing or fight and flight. The first validated instrument to measure feelings of desire/want for movement and rest, the CRAVE Scale (Cravings for Rest and Volitional Energy Expenditure) is already shedding light on the nature of these states. With these advances in theory, conceptual modeling and instrumentation, future investigations may explore the effects of desires and urges for movement and sedentary behavior in earnest.

## Introduction

The question of what motivates human movement, physical activity and sedentary behavior can trace its origin back to the time of the ancient Greeks. Aristotle was of the conviction that movement was the outcome of two faculties working together, practical reason and *desire* (Frijda, 2016; Shields, 2016; Nascimento, 2017). When weighing the contribution of each, he concludes that the “mind is not of itself sufficient to engender motion, but instead relies upon appetite” (Shields, 2016). Furthermore, he refines the roles of desire by stating its main function is to jumpstart movement. He states succinctly, “It is manifest, therefore, that what is called desire is the sort of faculty in the [mind] which initiates movement” (Shields, 2016). In other words, it is desire that actually “prick[s] practical intellect” and sets muscular movement in motion (Shields, 2016). Aristotle’s focus, therefore, was on the interaction of affective and cognitive factors to produce movement in the present moment.

In the last century, however, the emphasis has been on trying to understand the individual’s stable disposition for physical activity or exercise and promoting these behaviors on the time frame of the week, month or longer. Exercise behavior has typically been studied within the scope of several frameworks, including the mechanistic, social cognitive, socioecological, and humanistic/organismic paradigms (Rhodes et al., 2019). These later traditions are mainly rooted in cognitive-based perspectives which likely exaggerate the “capacity and willingness [of humans] to make rational decisions in order to achieve desired goals” (Brand and Ekkekakis, 2018). They tend to underplay or ignore affective factors, even though these are tantamount for the initiation and refinement of much of human behavior (Frijda, 2010; Ridderinkhof, 2017). For instance, only a few of these theories have considered the idea of desire or similar concepts. An exception seems to be Self-Determination Theory, (SDT), in which intrinsic motivation largely overlaps with craving/desire (Williams and Evans, 2014), and a lack of desire is a central feature of amotivation (Rhodes et al., 2019). Interventions based on cognitive constructs typically have low impact, as gauged by small effect sizes (Ekkekakis and Zenko, 2016), and they have “modest utility as mechanisms of behavior change” (Williams et al., 2019).

More recently, models have emerged that incorporate both conscious and unconscious, affective and deliberative factors affecting muscular movement in the present moment. Dual process theories, such as the Affect and Health Behavior Framework (AHBF), clearly articulate the role of desires and cravings in instigating various health behaviors, such as exercise and smoking (Williams and Evans, 2014; Williams et al., 2019). This model also highlights the central role of hedonic motivation. Williams and Evans (2014) state, “people typically crave/desire what they previously had a positive response to.” The central role of cravings in the formation and maintenance of “habit loops” has been the focus of a New York Times best-selling book (Atomic Habits) (Clear, 2018). Nevertheless, no formal theories exclusive to the domain of physical activity highlight the role of desires or urges for muscular movement (Brand and Ekkekakis, 2018; Rhodes et al., 2019; Williams et al., 2019).

Among those specific to movement, perhaps the theories most aligned with the idea of desires and urges are the Affective-Reflective Theory (ART) (Brand and Ekkekakis, 2018) and the model from Conroy and Berry (2017). Both of these models exist within the dual process framework and attempt to balance cognitive and affective processes to explain physical activity, and more specifically, exercise, at the moment activity is initiated (Brand and Ekkekakis, 2018). A relevant construct within both theories is the concept of the *action impulse*, which is the direct intermediary between automatic affective processes and movement behavior. These models do not specify, however, if impulses are conscious and felt. In motor control, the idea of desires for movement has been formally articulated as “wants,” which emanate from the inferior parietal lobule of the brain (Desmurget and Sirigu, 2012). In medicine, the concept of restless “urges” has been studied for decades (Garcia-Borreguero et al., 2011). The concept of “appetence” (i.e., strong and provoking desires for movement) has shed light on mechanisms of addiction (Ferreira et al., 2006). In musicology, the concept of “groove” describes the ability of music to generate feelings of urge to move (Matthews et al., 2019). There appears to be considerable overlap in the concepts of desires, wants, urges and related constructs, yet they seem to vary by the dimensions of magnitude, specificity to movement behavior, whether the desire is conscious and felt, as well as valence. However, all these concepts might commonly be characterized as *affectively-charged motivation states* (Kavanagh et al., 2005) *and associated feelings that signal a pressing need to approach or avoid a state of muscular movement (or, conversely a state of rest)*.

At this time, no previous analysis or review has attempted to evaluate, synthesize and expand on these motivation states, despite their prevalence in several intersecting literatures (Aristotle; Libet et al., 1983; Hausenblas and Downs, 2002; Gernigon et al., 2004; Reiss, 2004; Ferreira et al., 2006; Desmurget and Sirigu, 2012; Williams and Evans, 2014; Brand and Ekkekakis, 2018; Rhodes et al., 2019). In fact, most of the research on physical activity and cravings/desires investigates the efficacy of exercise to thwart cravings for other maladaptive behaviors, such as cigarette smoking or alcohol consumption (Ussher et al., 2008). Therefore, the objectives of this conceptual analysis are the following. (1) To describe the theoretical basis of desire as a primary motive of movement as well as sedentary behavior. (2) To provide evidence that such desires/urges exist and may be felt consciously. (3) To clarify terminology and the overlap between desires, urges, and cravings for movement. (4) Lastly, we aim to model relevant situations and emotion states associated with fluctuations in desires to move and rest and move/rest interactions. This paper is not intended to describe a multi-factor model of desires for rest/movement, such as ART or AHBF, but to more clearly highlight the important role of desires as they currently fit in established models (e.g., AHBF) or how they might naturally be included in similar models (e.g., ART). We also do not aim to describe in detail interactions between desires/urges and other factors relevant in active behaviors, such as goals, intentions and other cognitive-related constructs, which is beyond the scope of this current analysis.

## Discussion

### Drives, Tension, Reinforcement, and Reward

#### Hull and Drive Reduction Theory

Early studies of motivation conceptualized behavior as a function of instincts, drives, needs and tensions (Reiss, 2004) [see Ford (1992) for a review]. McDougall (1933) was instrumental in defining instinct as a function of “native human propensities” interacting with motor and cognitive “native abilities.” When propensities are stimulated by the environment they result in “an active tendency, a striving, an impulse, or drive toward some goal.” In Drive Reduction Theory, Hull (1943) described drives (energizers) as arising from innate physiological needs, such as the needs for water, food, air, and sexual activity. Physiological deprivation of these needs results in hunger, thirst, etc. and associated subjective feelings of tension (e.g., being hungry and thirsty). These felt tensions push those affected into action, and the amount of drive is proportional to the intensity of the resulting effort to satisfy the need. Drive reduction theory has poor performance in explaining complex human behavior, such as why humans willingly engage in strenuous and exploratory behavior that does not directly satisfy simple physiological needs (e.g., climb mountains). Drive reduction theory also includes a description of secondary drives, such as the drive for earning money, which are learned through conditioning (Weiner, 1982).

#### Kurt Lewin– The Dynamic Field, Tensions and Satiation

The work of Kurt Lewin and his contemporaries provided an important basis for the study of desires and urges. Lewin simply thought of human behavior as an interaction of the person with their environment, varying by the place of the person in an inner “life space” or dynamic field (Lewin, 1951; Marrow et al., 1969; Brand and Ekkekakis, 2018). This field incorporates a constellation of various needs, goals and motives - all changing with the situation – even on a moment by moment basis. In his Force Field analysis, driving and restraining forces act on a person to change behavior by propelling “locomotion” through a psychological field or environment, thus achieving an equilibrium. Lewin also described what he called “psychic tensions” in this field, which are “states of readiness or preparation for action” (Marrow et al., 1969) – not undesirable stress or strain. These emerge in response to a need, want or some other stimulus, manifest as “intention or desire” to carry out a specific task and are “released” when that task is completed (Marrow et al., 1969; Ridderinkhof, 2017). Less recognized is Lewin’s work on satiation of tensions with his protege, Anitra Karsten. She observed that desire to complete various movement tasks was indeed related to tension, and as a movement was repeated the tension dissipated and desire diminished - a state of satiation resulting in the behavior ending (Karsten, 1928). If the movement was forced to continue, the participant developed a great aversion to the task. Lewin saw this as a transition from psychological hunger to satiation to “oversaturation” and even related it to burnout – an “exhaustion of the will to work” (Lewin, 1928/2009; Soff, 2012). Importantly, satiation following constant repetition of a task was not due to muscular fatigue but simply a lack of desire.

#### Hedonic Pleasure and Reward

From a simple behaviorist perspective, the key to motivation is reward (Skinner, 1938; Skinner and Morse, 1958; Niv, 2007; Williams and Evans, 2014). In short, actions are repeated when reinforced – regardless as to whether this reinforcement occurs internally or from outside of the individual. There are two primary means of reward: positive reinforcement (providing a pleasurable stimulus) and negative reinforcement (taking away a negative stimulus). The strongest rewards follow those behaviors that result in both forms of reinforcement (Skinner, 1938). While the concept of the current study concerns human perceptions and behaviors, it is easier to demonstrate principles of reward with rodents. Imagine a rat who has been denied nourishment and is growing hungry. Providing it with highly palatable food will reduce the pangs of physical deprivation, which is negative reinforcement (Loewenstein, 1996; Tiggemann and Kemps, 2005). This food also provides an immediate positive reinforcement as it stimulates sensory responses that activate neural pleasure centers. If the food was acquired by pressing a lever (a muscular movement) this behavior will be highly reinforced (Skinner, 1938). Consequently, the rodent will continue to press the lever many times. Under conditions of severe hunger, this rodent will be motivated to contract its musculature with greater intensity (Salamone and Correa, 2002; Scheurink et al., 2010) in the effort to counter the aversive stimulus of hunger and in anticipation of a pleasurable reward. Such principles apply to humans as well. In neuroeconomics, the strength, persistence and vigor of muscular movements is considered a key predictor of what individuals value, find rewarding and prefer in their everyday choices (Shadmehr and Ahmed, 2020).

However, pressing a lever, like any type of physical activity, cannot be repeated indefinitely (Niv, 2007). The metabolic cost of movement (e.g., lactate) eventually sets in, resulting in painful and punishing sensations (O’Connor and Cook, 1999; Ekkekakis, 2013; Stults-Kolehmainen et al., 2016). Indeed, with growing fatigue, the punishment of movement becomes more aversive than hunger. Hence, movement is stopped, and rest occurs, which is yet another example of negative reinforcement as the cessation of muscular movement removes the negative stimulus. In this illustration, movement is merely instrumental (Salamone and Correa, 2002; Reiss, 2004); it is completed to acquire an outside source of reward (food) and remove hunger while avoiding excessive fatigue. Thus, from a behaviorist perspective, movement *itself* is not the source of pleasurable sensations, but it may be a source of considerable aversive sensations.

Is it possible that the act of movement itself may result in positive reinforcement? Many species will run purely for the sake of moving (Skinner and Morse, 1958; Garland et al., 2011; Roberts et al., 2012) and will even press a bar repeatedly to gain access to a running wheel (Collier and Hirsch, 1971; Belke and Pierce, 2014). However, it is debatable whether movement itself is naturally reinforcing in humans (Schultheiss and Wirth, 2008; Garland et al., 2011). Based on their empirical evidence, Cacioppo et al. (1993), Cabanac (2006a, b), and others (e.g., Schultheiss and Wirth, 2008) have argued that muscular movement must be reinforcing because it is the primary method of acquisition and consumption of many pleasurable stimuli, repeated many times over one’s life. In short, where movement is useful it must also be pleasant and wanted, at least occasionally. Furthermore, some human behaviors are fundamentally motivated even when there is no specific reward associated with them, such as exploration, perhaps because of the occasional discovery of a pleasurable, unconditioned stimulus (Schultheiss and Wirth, 2008). The extent to which these arguments are valid in a modern world, where machines can do both our labor and exploration, is uncertain. Nevertheless, it is generally accepted that voluntary physical activity may be agreeable (Garland et al., 2011; Boecker and Dishman, 2013), and there is an abundance of observations that some individuals even frolic, particularly children (Panksepp, 2006). That is, they move with joy and exuberance (Frijda, 1987; Panksepp, 2006).

In regards to structured exercise, the largest body of human research has centered on the potential of exercise to provide both immediate (mood and enjoyment) and delayed (body image) positive reinforcements, both directly and indirectly (e.g., social interactions) (Ekkekakis, 2013). Exercise increases vigor and feelings of positive well-being in both normal and clinical populations (Bartholomew et al., 2005). The phenomenon of a “runner’s high,” a state of euphoria during or following endurance exercise, is linked to opioid binding in prefrontal/orbitofrontal cortices of the brain (Dietrich and McDaniel, 2004; Boecker and Dishman, 2013). Exercise of almost any modality provides enhancements in affective tone, particularly during moderate intensity exercise and in the rebound period after strenuous exercise (Ekkekakis et al., 2011; Ekkekakis, 2013). Variability in feelings of pleasure during exercise (but not afterward) is a predictor of adherence to exercise programming (Williams et al., 2008; Rhodes and Kates, 2015), which seems to indicate that for some individuals, physical activity is rewarding and reinforcing for future behavior. Some individuals even “like it vigorous,” in other words, prefer a high level of intensity for their exercise (Ekkekakis et al., 2005) and find meaning and pleasure in the face of displeasure. Collectively, enjoyment, intrinsic motivation and affective attitudes about exercise appear to be key mediators in the relationship between affective responses to exercise and future activity behavior (Rhodes and Kates, 2015).

#### Physical Activity as a Negative Reinforcer

Movement is motivated not only by the optimization of pleasure, but by reducing displeasure (Cabanac, 2006a). What is lacking in the extant literature, however, is a thorough consideration of physical activity as a negative reinforcer – that movement may serve to alleviate tension (Williams and Evans, 2014). Some research exists concerning the relief of negative affective states (i.e., poor mood and distress) (Salmon, 2001; Stults-Kolehmainen and Sinha, 2014) and reduction in pain sensation (e.g., exercise-induced analgesia) (Bartholomew et al., 1996). However, these mood states have largely been considered as arising from an external source (e.g., work stress, social anxiety, etc.). That is, the activity itself is not tied to the *source* of negative mood. In these cases, exercise is no more or less effective than other means to improve mood (Thayer et al., 1994). As such, exercise is a choice no more compelling than relaxation practices, alcohol use, distraction (e.g., TV), and other forms of stress coping (Ingledew et al., 1996; Endrighi et al., 2016).

However, might it be that some of these negative states may be derived from the *lack* of physical activity? That is, could there be a drive for physical activity that requires some degree of activity to satisfy the need (Collier, 1970; Feige, 1976; Reiss, 2004; Schultheiss and Wirth, 2008; de Geus and de Moor, 2011; Kalupahana et al., 2011)? One might propose that all humans are “hard wired” for movement for: (a) instrumental reasons (e.g., foraging for food, seeking and building shelter, etc.), (b) for play, which helps to develop physical traits, develop social skills and improve affective tone (Panksepp, 2006), (c) for seeking out rewards, novel stimuli and new experiences (Schultheiss and Wirth, 2008; Panksepp and Biven, 2012; Frijda, 2016), (d) for acquisition and processing of information (Parker et al., 2020) and other reasons (Cabanac, 2006a, b). Aristotle concluded that desires to move and rest are the drivers which “prick” these behaviors in the moments before they are initiated (Aristotle; Shields, 2016; Nascimento, 2017). Other early literature [summarized by Ekkekakis (2013)] noted that humans have an “inherent propensity” or “drive for activity,” a need for stimulation or “susceptibility.” Many people even prefer electric shocks over total solitude (Wilson et al., 2014). More pertinently, these drives are *felt* as a “necessity of body exercise,” “volitional promptings” (Bain, 1855; Baldwin, 1891, 1894; Shirley, 1929; Hill, 1956; Finger and Mook, 1971) or tension, perhaps similar to appetite (Loewenstein, 1996; Rowland, 1998; Ferreira et al., 2006). Particularly under restrained conditions (i.e., prolonged sitting) humans feel “intense uneasiness or craving” or “pressing readiness.” Over 100 years ago, Williams James related the case of a girl that had a “morbid impulse” causing her to “walk, walk, walk” (James, 1907).

Almost anyone can identify with the discomfort of sitting for prolonged periods, feelings of being antsy, jittery, squirmy, restless and/or fidgety, and the relief provided by movement (Levine et al., 2005). Several slang terms are also associated with similar conditions and feelings, including being “cooped up,” “stir crazy,” or having “cabin fever.” Those with low back pain disorders fidget and shift their bodies, sometimes multiple times every minute, in order to relieve pressure and avoid pain (Dunk and Callaghan, 2010). However, systematic evidence for these behaviors are lacking in the current human literature. In research in the post-behaviorism era, such aversive sensations are generally linked to a limited number of phenomena, including urges associated with Restless Leg Syndrome (Garcia-Borreguero et al., 2011), akathisia (characterized by a compelling need to be in constant motion) (Iqbal et al., 2007), hyperactivity (Willerman, 1973; Scheurink et al., 2010), anorexia (Davis and Woodside, 2002; Scheurink et al., 2010), forced bed rest (Ishizaki et al., 2002), loss of playtime/recess (Jarrett et al., 1998), sudden decline in one’s usual exercise routine (Mondin et al., 1996), and exercise dependence/addiction (Hausenblas and Downs, 2002; Ferreira et al., 2006). Consequently, urges to move are well-documented in situations where such sensations are bothersome and unproductive.

#### Processes of Wanting

An alternative framework for understanding wants/desires for physical activity and rest is the incentive sensitization model (ISM) of rewarding behaviors (Robinson and Berridge, 1993; Berridge and Robinson, 1998; Roemmich et al., 2008; Boecker and Dishman, 2013). For any given pleasurable stimulus, the ISM proposes that there is both a hedonic *like* and an appetitive (motivational) *want* (Smith and Berridge, 2007), which vary in intensity from transient desires to cravings or urges. Likes and wants are typically tightly linked (as in the case of food or drugs), but these constructs differ in several important ways. First, there is evidence that they are controlled by different neurobiological systems – opiod for likes and dopaminergic for wants (Roemmich et al., 2008; Boecker and Dishman, 2013). Furthermore, *likes* and *wants* may become completely uncoupled, whereas in certain situations one may want to perform a particular behavior without necessarily liking it. For physical activity, this is most clearly relevant in the case of exercise dependence/addiction, in which individuals feel compelled to engage in physical activity even when it comes at great costs and is not enjoyable (Hausenblas and Downs, 2002; Ferreira et al., 2006).

The ISM also describes processes of wanting, including the prediction of when wants and desires may be whetted (stimulated), consummated and satiated. Wants are triggered by salient cues (irrespective of rewards), such as interoceptive sensations of tension, which may be amplified under conditions of physiological deprivation. In the case of physical activity, an example might be prolonged periods of unaccustomed sitting. Cues may also activate mental networks of associations (memory) to elicit urges (Rhodes et al., 2019). Furthermore, individuals become more sensitive to a reinforcing stimulus as it is repeated, causing the stimulus to be more salient and more attractive, leading to *wanting* the stimulus more. Flack et al. (2019a, b) have investigated exercise protocols lasting 3 months under this paradigm, and found that more frequent exercise, or exercise with more total volume (i.e., 300 kcal/day, 5 days/week) can increase the relative reinforcing value of exercise compared to sedentary activities in a group of untrained and overweight individuals. It is unknown if physical activity, outside of exercise, can become more rewarding and salient with repeated exposure, particularly for those who are typically inactive.

The ISM model has several drawbacks. First, it was developed in examination of rewarding substances like food or drugs. However, physical activity may differ from these substances in that: (a) the latter emanate from external sources, (b) consumption is highly tied, at least initially, to the experience of pleasure, while physical activity may not be, (c) they are subject to scarcity while movement typically is not and (d) movement is required for the acquisition of the former. Moreover, given the great utility of movement, it must be accomplished in day-to-day life, and thus is likely wanted, even if is not necessarily liked (Cabanac, 2006a, b). Consequently, liking and wanting of physical activity may be loosely coupled for most of the population. Lastly, this model underplays the role of affect, which is discordant from the well-established literature describing relationships between movement and emotion (Stults-Kolehmainen and Sinha, 2014; Ridderinkhof, 2017; Williams et al., 2019). Despite these downsides, the ISM model is valuable in emphasizing the importance of craving as an intermediary state prior to action, representing motivation and intent.

### Self-Determination Theory

In the view of Roberts (2012), the aforementioned theories of drive and behaviorism are “deterministic and mechanistic,” which “view humans as being passive.” Organismic theories, like Self-Determination Theory (SDT), and more specifically, the sub-theory of organismic integration theory, integrate and expand on the concept of human needs and drives, consider the person’s goals and feelings and evaluate the person in a social context (Deci and Ryan, 2000). The three basic psychological needs are competence, autonomy, and sense of relatedness. However, Deci and Ryan (1985) state that self-determination itself is the “capacity or fundamental need to choose and to have choices, rather than reinforcement contingencies, drives, or any other forces or pressures be the determinants of one’s actions.” People who are self-determined act upon fully internalized motivations, categorized into three distinct drives: intrinsic motivation to know, to accomplish and to experience stimulation (Vallerand et al., 1989; Vallerand, 1997). Performing movement for its own sake and for its enjoyment is considered intrinsic motivation (Reiss, 2004; Rhodes et al., 2019). Moreover, intrinsic motives can exert a powerful and long-lasting influence on exercise behaviors (Teixeira et al., 2012). Williams and Evans (2014) explicitly distinguish between sources of motivation (e.g., to experience stimulation) versus the affective charge attached to a motivation state. They conclude that, “intrinsic motivation is typically an affectively-charged motivation state that involves either craving/desire or fear”; however, they also recognize that movement is accomplished in the absence of strong desire. According to SDT, behavior is also influenced by extrinsic motives, such as tangible rewards (e.g., trophies), or wanting to be someone specific (e.g., a highly competitive athlete), which are farther down on the continuum of self-determination. Nevertheless, these also can result in strong desires, as in the case of having a strong, affectively-charged desire to perform sport because it is expected to result in strong social approval and the admiration of others (Williams and Evans, 2014). Ostensibly, one might have multiples desires at the same time, such as a desire for a reward (extrinsic) and a desire for movement itself (intrinsic). In this case, however, movement might be merely instrumental and secondary to the more pressing desire (Reiss, 2004). Amotivation, on the other hand, is a state of no motivation, or a near-total lack of desire or drive to perform a behavior. Consequently, SDT appears to be a complimentary theory, likely concordant with the idea of wants or urges for movement, that could be gleaned for information on how to broaden and categorize these desires.

### Dual Process Theories

#### Affect and Health Behavior Framework (AHBF)

The AHBF (Williams and Evans, 2014) outlines a dual process model in which learned automatic (unconscious) associations (A-system) and reflective/deliberative processes (R-system) work in concert to propel health-related behavior (Strack and Deutsch, 2004). In this model, “wanting” is considered *automatic motivation* as part of the A-system, along with the opposite construct, *dread.* Dread is a concept related to the emotion of fear – a motivational force that propels a person to move away from or avoid an aversive stimulus (Kringelbach and Kent, 2016). Williams and Evans (2014) distinguish these from desires, cravings and fear, which they label as *affectively-charged motivation* (Kavanagh et al., 2005). These are the product of conflict between automatic impulses (e.g., wanting and dread) and reflective intentions and goals (R-system) (Williams and Evans, 2014). Despite these divisions between desire, craving and wanting in their model, the authors seem to also categorize the former two as part of wanting and all of these, together with dread and fear, as part of affectively-charged motivation states (ACMS).

The model provides an understanding, though incomplete, of how an individual might have motivation states for physical activity. They note that, “people typically crave/desire what they previously had a positive affective response to,” which would seem to include physical activity. However, they later explain that these factors are typically only implicated with the experience of highly palatable food, sex, drugs, etc. (Williams and Evans, 2014). Physical activity is not considered a source of cravings/desire, but rather it is largely seen as a source of dread. In the example they provide, exercise is frequently and automatically associated with fatigue and pain, thus resulting in aversion as the automatic motivation. This restraining force may come into conflict, however, with a long-term intention of going outside to run. Whether an exercise action prevails is also influenced by competing behaviors (e.g., watching TV) and one’s current mood state and mental stress (Stults-Kolehmainen and Sinha, 2014). Despite some lack of clarity in this model, it provides an important advancement in the exploration of desires and cravings in physical activity research.

Williams et al. (2019) later revamped and extended the AHBF in their integrated framework (IF or AHBF-IF). Several improvements are observed in this model. First, it more clearly structures the relationships of all included factors, with paths starting with affect proper (specific mood and emotions), leading to motivation states and ending with behavior. It highlights the role that incidental affect can have on physical activity behavior (Lutz et al., 2010). Second, they collectively categorize wanting, desire, dread and aversion, so as to avoid the arbitrary divisions between these factors seen in the previous model. However, they relabel them as “hedonic motivation” as opposed to affectively-charged motivation states. A third strength of this revised model is that one can clearly delineate both the antecedents of affectively-charged motivation states (e.g., desires/urges) as well as their influence on behavior. Affectively-charged motivation states are seen in this model as proximally mediating the relationship between *affect processing* and behavior. As with other models discussed below, there is both an automatic and a reflective pathway, with affective processing and motivational processes occurring in both paths. Thus, the model supports goal-directed and purposeful motivation. Based on these improvements, more testable hypotheses may be formulated, and it is easier to generate examples of how desires might work in the real world. For instance, experiences of post-exercise affect (e.g., post-run euphoria) could result in an automatic association of running with pleasure. Anticipating this response again could lead to desires to run at another opportunity. On the other hand, the experience of inordinate work stress (incidental affect), resulting in poor mood, might activate automatic associations of exercise and excessive fatigue, resulting in an aversion to exercise, which squelches physical activity (Stults-Kolehmainen and Sinha, 2014). Overall, the AHBF-IF is an improved multi-factor model explaining how desires/urges may be generated and result in some movement behaviors – i.e., exercise.

Despite improvements, the AHBF-IF still has several limitations. A continued drawback with this model is that it is not specific to physical activity but generalized to all intentional health behaviors (e.g., smoking). Also, when considering physical activity, the models primarily seek to explain purposeful, structured exercise behavior, and it’s not clear if the model can explain the greater spectrum of physically active behaviors (i.e., task-specific movement, spontaneous movement, fidgeting). More significantly, cravings or desires *specifically for movement* are not considered in this model even though it opens the possibility that these exist. Because of the simplification and recategorization of affectively-charged motivation states (“hedonic motivation”), there remains ambiguity in the interaction between desires and dread for exercise behaviors. Furthermore, the model lacks a clear articulation on the role of restraining forces, such as the need or urge for rest and how these conflicts occur in the moment (Frijda, 2010). Finally, the model does not provide an explanation for how affectively-charged motivation states, such as desires, interact with goals and intentions.

#### Affective-Reflective Theory of Physical Inactivity and Exercise (ART)

Several recent dual process models have focused specifically on exercise behavior, such as the Affective Reflective Theory of physical inactivity and exercise (ART) from Brand and Ekkekakis (2018) and a similar model from Conroy and Berry (2017). These authors similarly hypothesize that movement is the product of the interplay between two systems: a type-1 automatic process and a type-2 process for reflective valuation (Conroy and Berry, 2017; Brand and Ekkekakis, 2018). They describe the conflict between an actual state and “desired state” (i.e., exercising) due to driving forces and restraining forces, as similar to the concept of tension systems from Lewin (1951). In their model, an exercise stimulus elicits a spontaneous affective response (i.e., pleasure/displeasure associated with the activity) through type-1 processing, resulting in an action impulse. Following this, slower type-2 processing is used to reflectively generate an action plan. The combination of the type-1 action impulse and type-2 action plan results in physical activity behavior. However, discordance can exist between the action impulse and the action plan. An example of this would be when a seated individual is exposed to an exercise stimulus (e.g., sees a person running), and immediately associates it with an aversive state (e.g., running is tiring – which is bad), which prompts the individual to remain sedentary. At this time, however, the individual also thinks about her/his doctor’s advice to exercise more frequently. In this case, Brand and Ekkekakis (2018) propose that the behavior that will follow depends on the availability of self-control resources, where a greater availability will result in the execution of the action plan (i.e., go exercise) instead of the action impulse to be sedentary.

There are limitations with the models from Brand and Ekkekakis (2018) and Conroy and Berry (2017). The first is that the concept of the action impulse is poorly defined, but seems to relate to a variety of other concepts, including: (a) Lewin’s description of psychic tension (Marrow et al., 1969), (b) the “prick” that was described by Aristotle (Aristotle; Shields, 2016; Nascimento, 2017), (c) the concept of “wants” as defined in motor control (Libet et al., 1983; Desmurget and Sirigu, 2012) or (d) it may be interchangeable with the concepts of *states of action readiness (SOAR), action tendency* (McDougall, 1933; Frijda, 1987; Frijda et al., 1989; Strack and Deutsch, 2004), *activation states* or a “specific motive state” in the description of *impulsive action* (Frijda et al., 1989, 2014; Frijda, 2010; Frijda, 2016). Nevertheless, the authors note that, “core affective valence may have a direct, immediate impact on behavior through behavioral urges” (Brand and Ekkekakis, 2018). The second problem is that this theory was created to explain the complex behaviors of exercise and regimented physical activities – as opposed to the greater spectrum of physically active behaviors, including spontaneous physical activity (Levine et al., 2005). The third issue is that the model represents sedentary behaviors as typically contrasting with physical activity; restraining forces pulling against propelling forces to alternate from one behavior to the other (e.g., flipping a single switch). However, rest and activity may not be in direct opposition. Instead, there may be restraining and propelling forces for both rest and movement acting simultaneously (e.g., two separate switches, or even two dials) (Beeler et al., 2012; Stults-Kolehmainen et al., 2020). The practical consequence of these limitations relates to intervening for muscular movement at the moment actions are being processed and how this might be modified or done flexibly based on desires for rest as well. Indeed, the purpose of these dual urges, working in concert, may be to “potentiate sets of action schemas with equifinality” for adaptive behavioral flexibility (Frijda, 2010).

#### Dynamical Model of Desire and Elaborate Intrusion Theory

Alternative multi-process models specifically highlight the powerful influences of wants/desires on human behavior. Hofmann and Van Dillen (2012) and Hofmann et al. (2012a, b) in their Dynamical Model of Desire draw on a diverse literature, defining desire as “a psychological state of motivation for a specific stimulus or experience that is anticipated to be rewarding [which] may or may not be consciously experienced” (Papies and Barsalou, 2015). This model also defines two routes by which desires can influence behavior: (a) an automatic, impulsive and unconscious route and (b) a route in which desires emerge into consciousness, become felt (e.g., have a subjective sense of wanting/feeling wants), interact with working memory and “hijack” cognitive processing. In the view of Kavanagh et al. (2005) in their Elaborated Intrusion Theory desire is “an affectively-charged cognitive event in which an object or activity that is associated with pleasure or relief of discomfort is in focal attention…it can be referred to as a conscious wish or urge to gain pleasure, relieve discomfort, or satisfy a want or to engage in consummatory behavior associated with these outcomes.” In this model, desire inherently involves cognitive processing and is often instigated by triggers (i.e., thoughts, cues, affect, and physical needs) that result in spontaneous, conscious and intrusive thoughts. Regardless of the definition or the specific factors in play, Hofmann et al. (2012a) have found that over 50% of a person’s waking hours are filled with various desires (Hofmann and Van Dillen, 2012). The most common desire is that for sleep, but desires abound for many rewarding stimuli: coffee, leisure, sex, and numerous other activities and objects (Hofmann et al., 2012b). Desire for muscular exertion is considered to be one of the most fundamental desires (Reiss, 2004). Unfortunately, only cravings for participation in sport activities has been systematically investigated (May et al., 2008; Hofmann et al., 2012a, b). In one exception, Katula et al. (2006) investigated the desire to be stronger and increase fitness. In this study, it was found that adding an empowering psychological intervention to a traditional strength training protocol increased the desire to gain strength in older adults.

#### Important Contributions From Motor Control

Up to this point, there has been little clarity on the issue of “action impulse” or “action readiness” and how they relate to the initiation of and wants for movement. Research in motor control appears to address this gap most adequately by investigating wants (e.g., often referred to as “intentions”) and urges at the level of simple movements (e.g., standing up, moving a finger). This work began with Libet et al. (1983), who asked participants to remember the moment they became aware of their want to move. This study was ground-breaking at the time because the data demonstrated that individuals’ neural preparation for movement (i.e., readiness potential) occurred before they became consciously aware of their want/intention to move. Matsuhashi and Hallett (2008) improved on flaws in Libet’s original study design and found that the intention to move goes through multiple layers of awareness and enters consciousness 1.42 s prior to actual movement initiation. The authors also found evidence of a “point of no return,” which occurs when the want/intention to move cannot be vetoed- an urge. Wants for movement have a neurophysiological basis and seem to originate in the supplementary motor cortex (SMA), pre-SMA, posterior parietal cortex (PPC), pre-motor area, motor cortex, intraparietal sulcus, and in the insular cortex (Lau et al., 2004; Fried et al., 2011; Desmurget and Sirigu, 2012; Li et al., 2015). Neuronal activity in the SMA precedes the conscious awareness of wanting/intention to move by 700 ms and predicts it with 80% accuracy (Fried et al., 2011). Furthermore, Desmurget and Sirigu (2012) found that the inferior parietal lobule is responsible for the preparatory “wanting to move,” while the mesial precentral area is responsible for the more powerful “urge to move.”. Collectively, the readiness potential and/or the conscious awareness of wanting to move might be referred to as the “action impulse.”

### Defining, Categorizing and Describing Desires/Urges to Move and Rest

**FIGURE 1:**
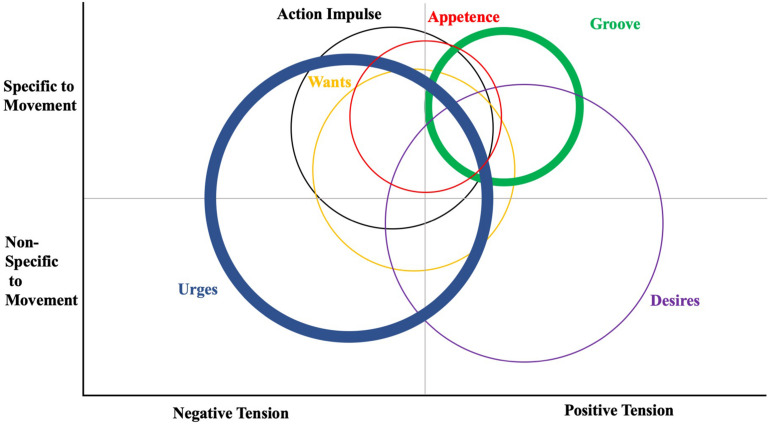
Venn diagram illustration of some affectively-charged motivation states (ACMS; urges, wants, appetence, groove, desires, and action impulses) as they vary by specificity to muscular movement and valence of the tension. Some concepts originated with movement (i.e., groove and appetence) while others are generally applicable to a multitude of reinforceable behaviors. Circle size denotes degree to which the state covers multiple quadrants. Thickness of circle line denotes prevalence of theme in current literature. [Not all ACMS constructs are included].

Based on a multitude of evidence, it is apparent at this juncture that desires/cravings for movement exist, but progress is still impeded by a lack of conceptual organization and instrumentation. The first issue is the abundance of related, yet distinctive, terminology – including the nomenclature above but also terms from Frijda and colleagues work on states of action readiness (SOAR), such as motor intention, longing, striving, “current concerns,” action tendency, urgency, control precedence, awareness of action readiness and “non-overt inclination” (Klinger, 1987; Strack and Deutsch, 2004; Pacherie, 2005; Elster, 2009; Frijda et al., 2014; Frijda, 2016; Ridderinkhof, 2017). Part of the confusion seems to stem from considerations of motivations states [e.g., urgency (Elster, 2009)], processes [e.g., “wanting,” (Berridge and Robinson, 1998)] and distinct, concrete felt perceptions and other unfelt forces (*a* “want”, *an* “urge”). To tackle this issue, the concepts of wants, desires, appetence, groove and the action impulse (i.e., action readiness) were all plotted on a field distinguishing them by apparent differences in valence (negative or positive tension) and their specificity to movement (see Figure 1). The concept of groove, for instance, is specific to a felt need to move, and is ostensibly conceived as a positive force or tension (Matthews et al., 2019). It is highly contextual to the influence of music, however. Urges to move, most cited in work on Restless Legs Syndrome, are highly specific in this context to muscular movement but are clearly gauged as a negative tension. Patients with Restless Leg Syndrome, other variants such as Restless Arm or Mouth Syndrome, and Tourette’s Syndrome report pressing, involuntary and bothersome urges to move and/or stretch that are often temporarily suppressed but eventually released (Cavanna and Nani, 2013; Jung et al., 2017; Ruppert, 2019). Appetence is highly specific to movement, but neutral in feeling in the same sense that appetite may be either present or lacking but distinct from the pangs of hunger (Ferreira et al., 2006). Wants have also been highly related to movement in the motor control literature, but usually are considered neutral in valence. Desires, on the other hand, have lacked specificity to movement and rest and have a positive connotation. Finally, it must be considered that constructs such as desire and want, while in this context highly concrete (e.g., they are discrete, observable and measurable) are typically used in a much more abstract and conditional sense (e.g., she wants to go for a walk after work). This makes completing literature reviews in the area difficult because keyword searches result in tens of thousands of irrelevant returns.

As mentioned above, there is a question about how desires/wants to move relate to desires for contrasting behaviors, such as being sedentary or resting. Thus far, the focus has been on motivation states for movement, but special attention is needed to elucidate whether rest-related wants should be conceived as a restraining force acting simply against movement or a separate dimension of wants interacting flexibly with those to move. In his early work, Frijda (Frijda, 1987; Frijda et al., 1989) characterized rest as a state involving an “absence of action readiness,” a feeling of not needing to do anything, rather than a separate dimension or system. However, there is reason to believe that rest and movement wants/urges operate in separate planes/continua and are not opposite ends of the same axis. First, as indicated above, other researchers have separated these into distinct desires, asking respondents to report whether they want to sleep, or rest or engage in movement activities (Reiss, 2004; Hofmann et al., 2012a, b). Indeed, sleep likely has its own drive (Hull, 1943). Second, such conceptualization of desires and rest as separate factors was demonstrated by a recent factor analysis (Stults-Kolehmainen et al., 2020). Finally, the idea of separate systems for rest and movement desires seems to be concordant with other similar work describing separate “go” and “no-go” (Beeler et al., 2012) and appetitive versus defensive systems (Frijda, 2010; Lang and Bradley, 2010). Thus, it appears reasonable that one can approach or avoid both desires for rest and movement separately (Frijda, 2010). In other words, rest and movement desires together do not correspond to a unidimensional approach/avoidance for movement.

**FIGURE 2:**
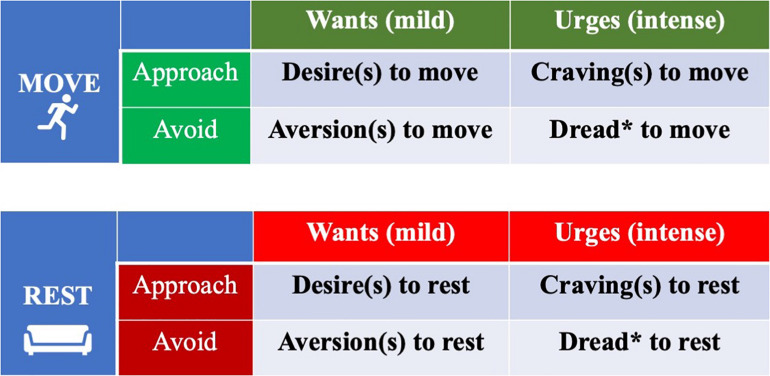
Want/urge motivation states specific to the domains of movement and inactivity behaviors: Approach/avoidance orientation vs. Intensity. This is a simple categorical model preceding the model described in Figure 3. *Singular and plural for impulses of dread (multiple instances of dread).

Conversely, it is proposed that there is approach/avoidance orientation for each system (rest and move) which corresponds to wants and aversions for each action (Cacioppo et al., 1993). These forces, in turn, vary by strength as well. One might consider the concept of dread as being more than the intense lack of urge to be in a state of movement/rest (i.e., a 0-point) but also an *active avoidance* of those states (Williams and Evans, 2014; Kringelbach and Kent, 2016). For instance, those with chronic low back pain and/or kinesiophobia exhibit fear and dread of movement and make attempts to actively avoid it, when possible, to prevent painful sensations (Barke et al., 2012). Consequently, it appears that a dimension of avoidance/approach, each of which varies by magnitude or level of activation/deactivation, is more appropriate than categorizing them by negative/positive valence (Watson et al., 1999; Rosenberg, 2009; Kemps et al., 2013; Williams et al., 2019). Furthermore, wants/desires, while typically affectively-charged, are independent from emotion (Kavanagh et al., 2005; Williams and Evans, 2014; Williams et al., 2019). In contrast, wants/desires appear to often be triggered by and result in various emotion states (Frijda et al., 1989). Regarding magnitude, desires/wants ostensibly can range from very weak to very strong. In motor control, strong wants are labeled as “urges,” and importantly, urges are closer to the actual manifestation of movement than wants (Desmurget and Sirigu, 2012). Figure 2 provides an intermediary categorization how wants, desires, urges and cravings, the most described motivation states, might be conceptually organized to explain movement and rest behaviors. It explicitly divides move and rest wants/urges into separate categorizations. A substantial shortcoming of the simple categorization of motivation states in Figure 2 is that it does not consider how desires for rest and movement can interact to produce flexible and adaptive behavior.

### The WANT Model

**FIGURE 3:**
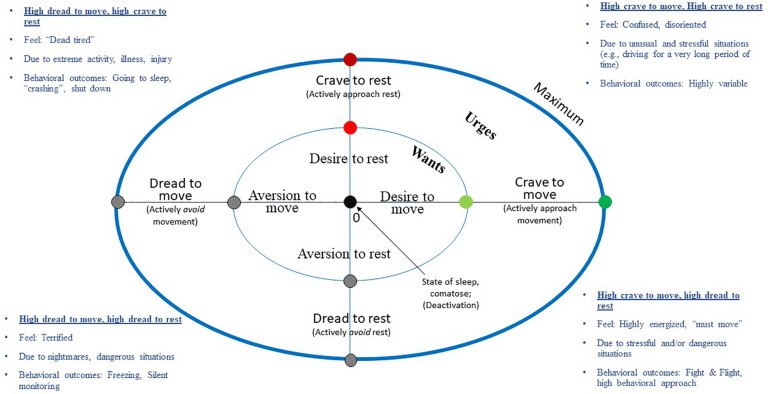
Wants and aversions in neuromuscular tasks (WANT) circumplex model of affectively-charged motivation states (ACMS, i.e., wants, urges, aversions, and dread) to move and rest. Interactions between these factors are associated with a variety of situations, emotions, and psychological phenomena.

How desires to move and rest interact might be best visualized in an orthogonal perspective. Given the logic above outlining separate dimensions or systems for move and rest, it’s proposed that one might occasionally occupy conditions in which one is high in both desires to move and rest – as well as low in both. The same may be true for avoidance orientation (e.g., high in the need to avoid both movement and rest, i.e., dread) (Jean-Richard-dit-Bressel et al., 2019). Figure 3 plots the WANT (Wants and Aversions for Neuromuscular Tasks) model. This is a descriptive, circumplex model (Guttman, 1954; Acton and Revelle, 2002) of affectively-charged motivation states (i.e., ACMS; desire, urges, aversions and dread) to move and rest, the continua of which are positioned orthogonally from each other. Importantly, and unlike other models, both desire and dread are modeled on the same continuum as opposed to being separate constructs (Williams and Evans, 2014; Williams et al., 2019).

The WANT model was designed to plot, categorize, and help to describe potential situations in which desires/urges to move/rest may occur, as well as associated emotional phenomena that may be generated in those circumstances. For instance, simultaneously experiencing very high rest and very high move wants/urges might occur in situations, such as: (a) having just won a competition and wanting to celebrate, but also being physically exhausted, (b) being torn between the need to workout or rest/have a meal, (c) suddenly becoming injured in the middle of competition, (d) overtraining, or other situations in which one might feel conflicted. Being low in both move and rest wants/urges, conversely, would be closer to the intercept of these axes, a state of deactivation that might be similar to a state of depression (Frijda, 2016) or possibly a meditative state of mindfulness and stillness. The WANT model also delineates avoidant motivation states characterized by feelings of *not* wanting to rest to move (diswants), in other words, having an aversion or dread. Relevant emotional outcomes in these quadrants include a variety of stressor states, like fight or flight (Frijda et al., 1989). Conversely, one might experience freezing behavior in the face of danger, which might be characterized as motivational states high in dread for both movement and rest (Frijda, 2016). Frijda (2016) argues that in such situations, there is no action readiness and individuals face “motivational null states” because “no meaningful action can be conceived.”

The WANT model incorporates several advances. First, it explicitly describes affectively-charged motivation states (ACMS) along several dimensions: (a) move vs. rest axes, (b) approach (desire/urge) vs. avoidance (aversion/dread) orientation and (c) relative strength of desire (inner vs. outer circle symbolizing want vs. urge strength) with a 0 point and a hypothetical maximum. This allows the reader to determine relations more easily among variables. There is also predictive value to this taxonomy. As suggestive above, a score high in move and low in rest might be predictive of a highly energized state of action readiness for action. Frijda (1987) describes excitement as “impulse toward restless movement, with frequent changes in direction” and not being able to sit still (Frijda et al., 1989). Likewise, exuberance is “impulse toward enhanced movement scope and movement abundance” with those selecting this emotion reporting, “I wanted to move, be exuberant, sing, jump, undertake things” (Frijda, 1987; Frijda et al., 1989). Therefore, the model permits categorization of various situations, emotions and feelings as described above by quadrant (e.g., high move/low rest; high move/high rest). One can also map transitions, both sudden and gradual, in move/rest desires and placement in “fuzzy” situations (e.g., feeling really tired but also relying on one’s body to get home after work) (Guttman, 1954; Acton and Revelle, 2002).

The utility of the WANT model to help formulate testable hypotheses or predict *future* states or behaviors is yet to be determined. How specific desires/urges (or combination of desires) interact with other factors to ultimately drive behavior is not modeled. Pacherie (2005), Frijda et al. (2014), and Frijda (2016) point to the fact that desires and states of action readiness (SOAR) do not absolutely determine behavior. Rather, desires are highly flexible in their behavioral outcome since many different actions can result from the consummation (or not) of desire (Frijda, 2016). Such flexibility is likely advantageous and adaptive. This being said, the relative strength of competing urges plays a part in behavioral choice (Hofmann et al., 2012a, b). Deliberative factors also can act on specific desires to enhance or minimize their impact (Hofmann et al., 2015). Lewin’s force field analysis seems to suggest that a multitude of forces work in tandem to help an individual achieve a state of equilibrium (Marrow et al., 1969; Brand and Ekkekakis, 2018), and this varies by a variety of individual needs, motives and situational factors which are beyond the scope of this model to include. From a neuropsychological perspective, how one behaves might primarily be a function of dopamine regulation, as in the “go” and “no go” model (Beeler et al., 2012). While the WANT model is not high in predictive value, it is intended to be helpful for understanding the nature of desires/urges, which is needed before adequate path models can be created.

### Future Research

Research on desires or urges to move and rest is still in formation, and many avenues of research exist to realize its potential. To move forward, the following 10 areas should be considered.

(1) While some progress has been made in defining desires and urges, how they relate to each other, and how they may influence physically active or sedentary behaviors, there are no systematic literature reviews in this area of inquiry.
(2) The fundamental nature and descriptive quality of desires/urges to move still needs clarification. One basic issue relates to conscious awareness of desires/urges to move and how they are experienced or felt (Frijda et al., 1989; Hofmann et al., 2015). Are they felt as positive or negative, intrusive, and/or unwanted? Do they vary from young to old age? How frequently do they occur and how quickly can they change (Gernigon et al., 2004)? Are they more prominent during the acquisition or solidification of physical activity habits (Clear, 2018; Greenwood and Fleshner, 2019)?
(3) The WANT model (Figure 3), describing the orthogonal nature of desires to move and rest needs testing and empirical validation (Acton and Revelle, 2002), the beginning of which is described in a recent paper (Stults-Kolehmainen et al., 2020). One drawback is that the model does not explicitly include an additional dimension of affect (e.g., pleasure/displeasure), chronicity (i.e., a single urge versus constant craving), or effect on motor behavior, which may necessitate refinements of the model.
(4) Is there a threshold of want/urge magnitude to initiate movement, as suggested by the motor control research (Desmurget and Sirigu, 2012), and how does this relate to conscious awareness of the desire or urge (Libet et al., 1983)? The definition of “maximum” should also be clarified, whether that is defined psychometrically (e.g., feel “more than ever”), cognitively (i.e., the urge dominates thoughts) or behaviorally (e.g., motor actions have been initiated).
(5) There is an obvious lack of a model, specific to movement and rest, to expound on the antecedents of desire and the varying impact on behavior, which could help to create testable hypotheses for future investigations. Such a model would be best fashioned in light of the multiple disciplines that have a shared interest in desires/urges to move, such as exercise psychology, motor control, clinical medicine and psychiatry (Hausenblas and Downs, 2002; Ferreira et al., 2006; Iqbal et al., 2007; Garcia-Borreguero et al., 2011; Desmurget and Sirigu, 2012; Williams and Evans, 2014). Another possibility is that models, such as AHBF (Williams et al., 2019), could be slightly modified based on the observances from the WANT model (Figure 3). Such a model may consider the bi-directional nature of wants and urges with affect, emotion and mood. Can unsatisfied and unremitting urges and cravings exponentiate through worsening mood in a cyclic fashion? Understanding the complex relationships between these factors has significant implications for movement-based interventions.
(6) The processes primarily described here predominantly relate to automatic and impulsive processes, but much needs to be done to formulate how desires/urges interact with deliberative (reflective) processes. Certainly, impulses can be overridden by higher order cognition (e.g., goals) (Stults-Kolehmainen et al., 2020) and desires can relate directly to goals (Gernigon et al., 2004). Feige (1976) suggests that motivation for physical activity is a 5-level hierarchy, with drives to be active forming the foundation and goals and values in the highest level. These interactions have already been briefly detailed by theories, such as ART and AHBF, but also by: (a) the Model of Goal Directed Behavior, in which desires interact with intentions to pursue a goal (Dholakia, 2015), (b) the Grounded Theory of Desire and Motivated Behavior, in which environmental cues can spark memories, cognitions and mental re-enactments, which generate desires (Papies et al., 2020), and (c) the Elaborated Intrusion Theory, in which suppression of desire-related thoughts can lead to stronger desires (Andrade et al., 2015). Interactions between desires/urges and deliberative processes are also prominent in research on clashes between these constructs, as in work on: (a) want-“should” conflicts (Bitterly et al., 2015), (b) goal-desire conflicts (Hofmann et al., 2015), and (c) desires, reasoning and self-regulatory failure (deRidder et al., 2015). To summarize, there appears to be bi-directional and dynamic relationships between desires and goals; desires can hijack thoughts, be diminished by thoughts, work with thoughts toward a goal or undermine a goal.
(7) One question yet to be resolved is concerning a hierarchical typology for desires to move. More specifically, how do we distinguish between the human need to move for the sake of movement (primary desires) or simply to acquire something or accomplish some other task (secondary desires) (Reiss, 2004)? For instance, it is a common sensation to feel the urge to move when needing to urinate (Coyne et al., 2012), but this urge is secondary to the primary motive. More information is needed on how wants to move relate to wants for structured exercise or “working out,” getting stronger, becoming leaner, etc., whether complimentary or not (Katula et al., 2006).
(8) Up to this point, there was a lack of validated instruments to assess desires/urges for movement and sedentary behavior. However, this gap was just recently addressed with the creation of the CRAVE (Cravings for Rest and Volitional Energy Expenditure) scale to measure desires/wants for movement and sedentary behavior (Stults-Kolehmainen et al., 2020). This novel instrument must be further investigated and validated under different conditions (e.g., prolonged sedentary behavior, different psychological moods, and different exercise activities).
(9) Future investigations should also be sensitive to linguistic and cultural differences. For instance, how desires, wants, urges and cravings translate in Portuguese might correspond to the words desejo or vontade (desire), querere (want), impulso (urge), necessidade or $\overset{⌢}{\text{a}}$nsia (craving) or even saudades – intense desires or longings for almost anything that is missing (Neto and Mullet, 2014).
(10) Utilize the concepts of desires/urges to move to explain other phenomena of interest. We focus on two examples. First, desires/urges for movement and rest might moderate the relationship between psychological stress and physical activity (Stults-Kolehmainen and Sinha, 2014), which seems to fit within the tenets of the AHBF-IF model (Williams et al., 2019). Second, the development of this concept may also be expanded to inform how energy availability (e.g., overfeeding or a deficit), and more generally nutrition, affects urges to move or be sedentary. For instance, a surplus of calories might result in altered desires to move for some people, which then may influence variations in non-exercise activity thermogenesis (NEAT), particularly spontaneous physical activity (i.e., fidgeting, posture adjustments) (Levine et al., 1999; Rosenbaum and Leibel, 2016) and other compensatory behaviors (King et al., 2007).

Such considerations provide fodder for a multiplicity of future investigations.

### Practical Implications

Understanding the underpinnings that lead to desires/urges for movement or rest may have vast practical implications in fields such as exercise science, motor control, performance, and physical therapy. Unfortunately, up until this time motivation states for movement were overlooked, considered irrelevant or categorized as a nuisance factor other than a real point of possible intervention. For instance, Williams et al. (2019) did not identify motivation states as a possible route of intervention in their integrated framework. However, given the potential stated above it is reasonable to consider methods that can enhance the desire to move. There are six general approaches relating to desires and urges: (1) To improve movement wants, modifying the reward value of exercise by making it less punishing and/or more pleasurable (increasing the “like”), (2) varying physical activity and exercise to result in less rapid satiation of desires, (3) modifying environmental and situational conditions to either ramp up motivation states to move and/or possibly dampen motivation states for rest, (4) modulating psychological attention to these desires so individuals might be more sensitive/attuned to desires, both noticing them when they occur and acting on them and (5) “nudging” people in response to these noticed desires/cravings, particularly with cues (Thaler and Sunstein, 2008; Hofmann et al., 2015), and (6) taking advantage of urges/desires for other rewarding behaviors to encourage development of desires to move. Regarding this last point, some work is already being done on gamifying movement, making games contingent on moving (e.g., Pokemon) to increase the reward value of movement (Kaczmarek et al., 2017).

It seems sensible to start with this approach of modifying exercise. Can exercise be modulated to make it more rewarding, and thus result in greater “wants”? Exercise can be modified to increase enjoyment by focusing on preferences (Stults-Kolehmainen et al., 2013; Busch et al., 2016) or reduce punishing aspects of exercise, like avoiding eccentric contractions (Kerksick et al., 2009) or excessive buildup of lactate and fatigue (e.g., minimized with sprint interval training – SIT) (Benitez Flores et al., 2018; de Sousa et al., 2018). It is likely important to avoid sudden, large increases in novel physical activities that result in excessive muscle damage and soreness, which are associated with decreases in physical activity (Proske, 2005; Stults-Kolehmainen et al., 2014) and negative shifts in mood (O’Connor et al., 1991; Stults-Kolehmainen and Bartholomew, 2012). We could also modify conditions to promote desires so that desires/urges are felt more frequently and/or with greater intensity. For instance, music often leads to muscular movement as humans can sense desires to move in response to a beat (i.e., groove), and musical cues appear to elicit neural firing (Levitin et al., 2018). It is likely that humans can even form internal representations of a beat so that anticipatory movement can occur in preparation for music, which is coordinated by the cerebellum, the supplementary motor area and the pre-motor cortex (Levitin et al., 2018). Motivational videos and other visual images (i.e., highly fit individuals, major sport feats, etc.) may also stimulate improved movement motivation and performance (Barwood et al., 2009; Cope et al., 2018). Environmental conditions, particularly daylight, can have a significant impact on levels of physical activity (Tucker and Gilliland, 2007). While weather cannot be changed, contingency plans can be put into place to modulate desires to move, and thus behavior, in response to varying conditions. One might imagine that it is possible to nearly perfect exercise conditions (e.g., ambient lighting, exercise-related imagery, diet, music, social interactions, acceptable stimulants, like caffeine) to facilitate greater muscular movement. Undoubtedly, coaches, commercial gyms and others already engage in such efforts to create attractive environmental and motivational climates to spur movement and its enjoyment (Shaulov and Lufi, 2009; Bird and Karageorghis, 2020).

Regarding approach 4, a large literature, starting with Libet et al. (1983) demonstrates that even healthy and well-functioning individuals can be trained to pay attention to urges for movement (Lau et al., 2004). Unfortunately, protocols in these studies have not been used in attempts to facilitate greater movement but were designed to investigate the control of movement. Perhaps simply asking someone about their desires/wants to move can instigate motivation states for movement. In populations suffering from addiction or stress, mindfulness meditation has been used to help individuals sense desires and then “ride the wave,” interpreting appetitive stimuli as “mere mental events” as a method to cope with dysfunctional urges (Braun et al., 2012; Papies et al., 2015, 2020; Jastreboff et al., 2018). In line with these advances, perhaps a method, such as mindfulness and/or vivid imagery, could be developed or modified to promote greater movement (Kavanagh et al., 2005). This might involve generating desires/urges to move or simply paying attention and “listening” to them, thus bringing them fully into conscious awareness, gauging them, and consequently acting or consciously not acting on them (Devereaux, 2013; Naves-Bittencourt et al., 2015; Stults-Kolehmainen et al., 2015; Keesman et al., 2016; Renner et al., 2019; Papies et al., 2020). This approach seems promising but is still theoretical, and its efficacy is unknown.

## Conclusion

We conclude that there is a conceptual basis for desires and urges to motivate human movement and sedentary behavior. Such understanding is still in its infancy, particularly because of numerous similar concepts in literatures isolated from each other and a corresponding lack of coherence in definitions. Nevertheless, desires and wants for movement appear to be common constructs across multiple relevant theories. The current investigation conceptualizes physical activity primarily as a negative reinforcer. Humans likely have a “need for activity” that varies in intensity across the population (Rowland, 1998; de Geus and de Moor, 2011), is not simply a lack of “need to rest,” and may be felt as tension when unsatisfied. These salient, internal cues may elicit wants or desires to rest and move, in other words, fluctuating states of motivation to either expend energy or be sedentary. In some situations, or for individuals with certain conditions, desires for activity may be experienced as urges or even cravings (Ferreira et al., 2006). This manuscript describes the WANT (Wants and Aversions for Neuromuscular Tasks) model, a circumplex model of wants and urges for movement and rest, where these factors are placed orthogonally. This heuristic might help to inform how movement and rest wants might be observed in a variety of situations. Unfortunately, no models specific to movement and rest exist to explain both how desires are precipitated and exert influence. Such models should expand beyond the automatic and impulsive level of processing predominately described in this manuscript to include interactions between desires/urges and reflective factors. The AHBF-IF is, perhaps, closest to this proposed model. Up to this point, instrumentation to measure desire has been lacking. However, the recent validation of the CRAVE Scale is an example of an advancement that might facilitate further understanding of why and how changes in movement and sedentary behavior occur across the day. Data generated in this regard may help to understand daily fluctuations in energy expenditure in both healthy, formal functioning populations as well as clinical populations where perception and manifestation of muscular movement is problematic. It is our desire that this conceptual analysis will provide a starting point for future investigations.

## Author Contributions

MS-K, MB, RS, JB, TG, GA, and PM (in order by contribution) developed the conceptual analysis, wrote the manuscript, and designed and created the figures. All authors contributed to the article and approved the final submitted version.

## Conflict of Interest

The authors declare that the research was conducted in the absence of any commercial or financial relationships that could be construed as a potential conflict of interest.
